# The deltopectoral flap in full-thickness cheek defect: A case report

**DOI:** 10.1016/j.amsu.2021.01.025

**Published:** 2021-01-18

**Authors:** Ouassime Kerdoud, Rachid Aloua, Faiçal Slimani

**Affiliations:** a Faculty of Medicine and Pharmacy, Hassan II University of Casablanca, B.P, 5696, Casablanca, Morocco; b Oral and Maxillofacial Surgery Department, CHU Ibn Rochd, B.P, 2698, Casablanca, Morocco

**Keywords:** Cheek, Reconstruction, Restoration, Deltopectoral flap, Squamous cell carcinoma

## Abstract

**Introduction:**

Defects caused after tumor resection should be closed with flaps that match the neighboring cheek's skin.

**Presentation of case:**

the authors report a patient diagnosed with squamous cell carcinoma and its management. A 50-year-old man patient presented with a painless slow swelling in the left cheek, which had increased in size in the last four months, tobacco smoking, and alcohol intake for 15 years. Clinical examination revealed left cheek swelling without any lymph nodes at the palpation.

**Discussion:**

Reconstruction of the full-thickness is a real challenge. The deltopectoral flap offers several advantages despite the increasing use of microvascular reconstruction; technically is a simple and reliable flap that is preferred for the reconstruction of large through-and-through defects after resection of oral carcinoma. Preoperative planning of flap and early recognition of issues can avoid postoperative complications.

**Conclusion:**

This reconstruction technique was demonstrated in large, full-thickness defects involving the cheek.

## Highlights

• Reconstruction of through-and-through defects of the head and neck is a challenge.
• The deltopectoral flap is used despite the increasing use of microvascular reconstruction.
• Technically is a simple and reliable flap that is preferred for the reconstruction of large defects after resection of oral carcinoma.
• The main treatment of surgical management of head and neck tumors is to achieve a satisfactory functional and aesthetic results.

## 1 Introduction

The deltopectoral (DP) flap remains a viable alternative if extensive resection is also beneficial for the reconstruction of the cheek, offering sufficient transferable cutaneous tissue [1].

The deltopectoral (DP) flap may be useful in reconstructing the entire cheek by transferring cutaneous tissue [2,3]. Besides, the cutaneous layer of the flap can tolerate the rotational movement allows placement in varying directions.

The authors report the case of extensive resection of squamous cell carcinoma (SCC) in a 50-year-old man and deltopectoral flap reconstruction which offer good functional and aesthetic results.

### 1.1 Case report

Our work is a single case report and has been reported in line with the SCARE criteria [4].

A 50-year-old man complained of a painless slow swelling in the left cheek, which had increased in size in the last four months, tobacco smoking, and alcohol intake for 15 years, without any chemical exposure. There was no history of local trauma, radiotherapy, HPV infections, or family history of malignancy. He was referred to our department's consultation for specialized care. No other personal or family history was raised during the patient interrogation.

**Fig. 1:**
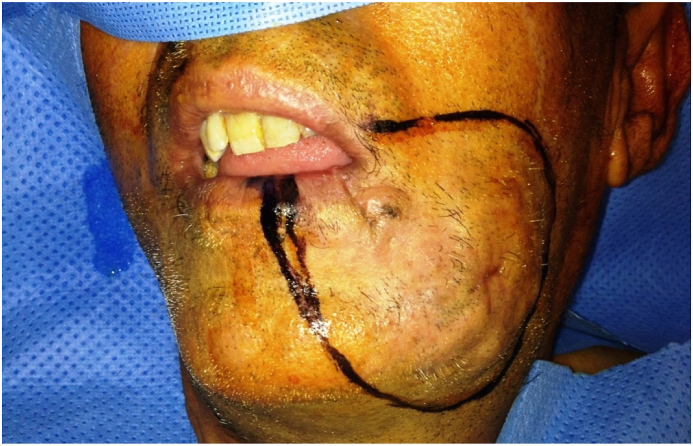
Preoperative time (surgical margins designing of squamous cell carcinoma).

Clinical examination showed painless swelling in the left cheek, a 3 × 3 cm mass presenting with irregular borders (Fig. 1).

There were no palpable neck lymph nodes on the left side.

A biopsy of surgical material was performed, showing a well-differentiated squamous cell carcinoma.

The definitive diagnosis of malignancy was made; metastatic investigations were performed, including chest radiography, abdominal echography, PET scan. The results of these exams were normal.

The computed tomography (CT) was performed and revealed a 4 × 4 cm mass along the cheek, and the alveolar bone was eroded.

**Fig. 2:**
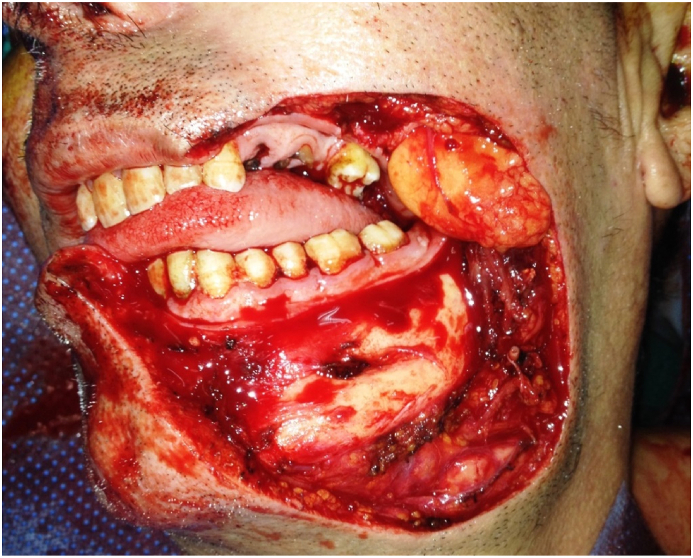
Full-thickness cheek resection.

**Fig. 3:**
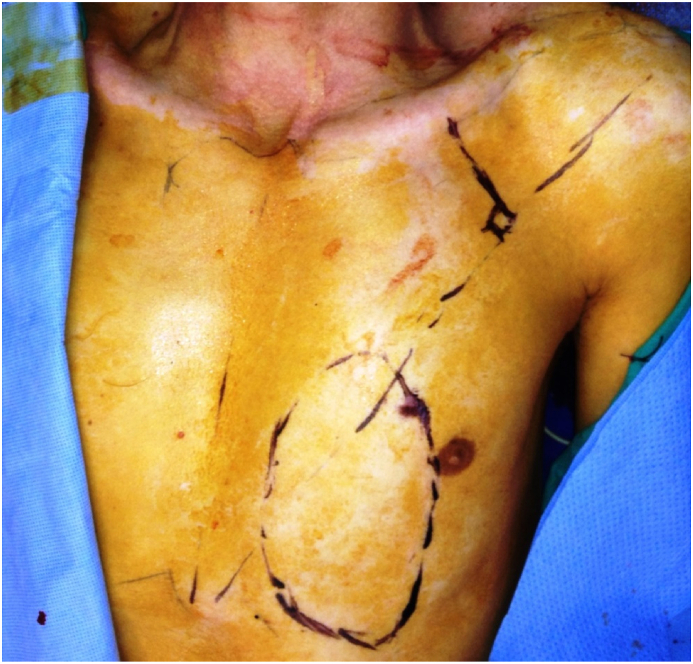
The deltopectoral (DP) flap designed.

**Fig. 4:**
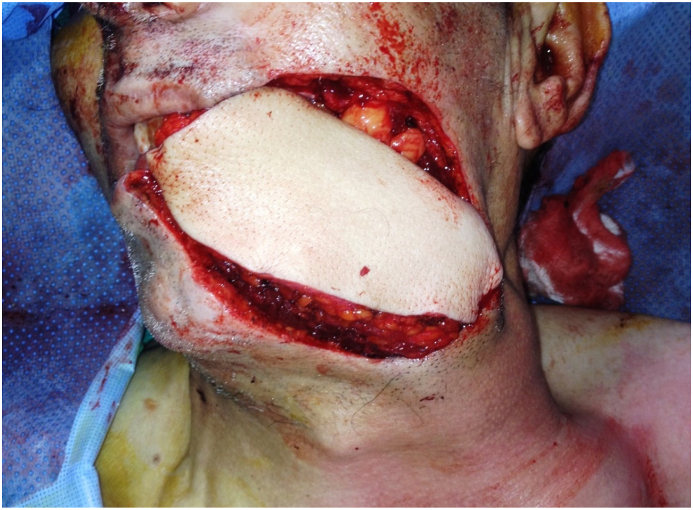
The deltopectoral (DP) flap in position.

Based on the positive medical history and clinical examination, surgery was indicated and performed by the chief professor of our department who has 15 years of operative experience. The surgical procedure had the aim of the restoration of the anatomic landmarks after a large excision of the tumor and reconstruction of full-thickness cheek defect with a deltopectoral flap under general anesthesia. (Fig. 2, Fig. 3, Fig. 4).

Duration of surgery: 120 min; estimation of blood loss: 350 ml; duration of hospital stay: 4 days.

The patient received amoxicillin/clavulanic acid 1g twice daily and antalgics for 8 days.

Post-operative histopathology confirmed SCC proliferation (Fig. 6).

The multidisciplinary team deciding on the treatment options included surgeons, oncologists, radiotherapists, ENT surgeons, radiologists. The decision was made to treat the patient with the surgical approach with postoperative radiotherapy (PORT) and postoperative chemotherapy (POCRT). Considering the patient's age, general history, the size of the tumor, the prognosis was poor.

Postoperative periods were favorable; the scar was clean and non-inflammatory.

Routine follows up 3, 6, and 12 months later showed no signs of recurrence.

## 2 Discussion

The deltopectoral (DP) flap is one of the most frequent and valuable flaps used in cheek reconstruction. The use of the deltopectoral flap is one of the flaps which it is easy to harvest.

The intrinsic and extrinsic factors are incriminated in the development of malign tumors that are well described in the literature, especially the contribution of smoking habits, actinic radiation, HPV, and chronic infections [5].

The most common malignant tumors of the face are basal cell carcinoma, squamous cell carcinoma, and melanoma [6]. The unfortunate fact of the facial skin is that it is impossible to have large resection without significantly affecting vital neighboring structures. The cancers of the head and neck must be removed optimally.

**Fig. 5:**
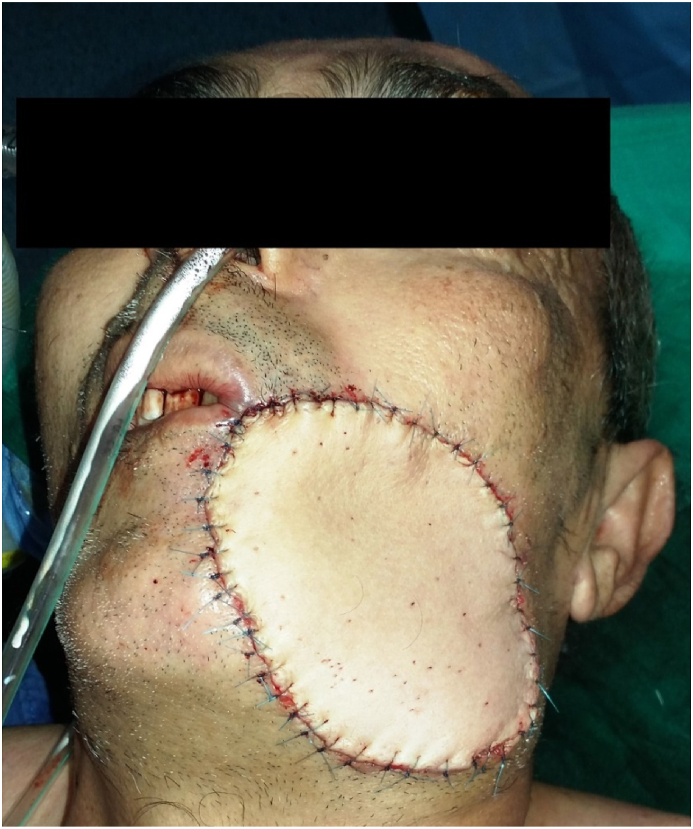
End of surgery.

The main treatment of surgical management of head and neck tumors is to achieve satisfactory functional and aesthetic results (Fig. 5); reconstruction of large defects requires a careful restoration of all missing components, adequate texture matching, and functional restoration and the contralateral cheek which serves as a template in reconstruction modalities [7]. The decision-making and the choice of the flap are made according to the size of the tumor and defect, location, relationship to adjacent structures, and existing skin tension lines [8].

**Fig. 6:**
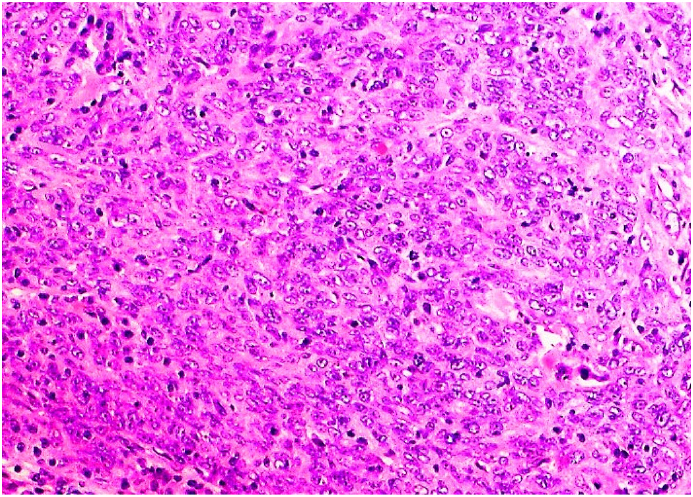
Squamous cell carcinoma (histopathology).

Surgical resection of cheek tumors frequently results in a full-thickness defect with direct exposure of the oral cavity and may lead to unfavorable aesthetic and functional problems for the patient [9]. There are several options for the reconstruction of cheek defects, including local or free flaps [10]. Reconstruction of cheek soft tissue defects after tumor resection is challenging because it is necessary to reconstruct both the intraoral anatomy and skin out-layer [11]. In the case study, the main goal in the cheek reconstruction to achieve: maintenance of oral competence, sufficient oral access, restoration of the anatomic landmarks and facial subunits, adequate tissue match in terms of color, maintenance of lips relation.

The deltopectoral flap has the advantage to offer a similar skin color and texture on the cheek versus the free flap may have a success rate, but patient satisfaction is relatively low due to the difference in the neighboring cheek's skin [12].

Preoperative planning of flap and early recognition of issues can avoid postoperative complications. A critical complication in cheek reconstruction is ischemia, which will lead to necrosis of the flap. There is an increased risk of flap failure with smokers [13].

## 3 Conclusion

The deltopectoral flap is one of the most common and valuable flaps used in cheek reconstruction. Reconstruction of cheek soft tissue defects after tumor resection is a challenge. The decision and choice of the flap are based on various factors such the skin color, the rate of success, the size of the defect, and the maintenance of oral competence.

## Provenance and peer review

Not commissioned, externally peer-reviewed.

## Ethical approval

Written informed consent was obtained from the patient for publication of this case report and accompanying images. A copy of the written consent is available for review by the Editor-in-Chief of this journal on request.

## Source of funding

The authors declared that this study has received no financial support.

## Author contribution

Ouassime kerdoud: Corresponding author writing the paper.

Rachid Aloua: writing the paper.

Faiçal Slimani: Correction of the paper.

## Registration of research studies

1 Name of the registry: researchregistry
2 Unique Identifying number or registration ID: 6312
3 Hyperlink to your specific registration (must be publicly accessible and will be checked):

## Guarantor

OUASSIME KERDOUD.

## Declaration of competing interest

Authors of this article have no conflict or competing interests. All of the authors approved the final version of the manuscript.
